# 6-shogaol, a neuroactive compound of ginger (*jahe gajah*) induced neuritogenic activity via NGF responsive pathways in PC-12 cells

**DOI:** 10.1186/s12906-017-1837-6

**Published:** 2017-06-24

**Authors:** Syntyche Ling Sing Seow, Sok Lai Hong, Guan Serm Lee, Sri Nurestri Abd Malek, Vikineswary Sabaratnam

**Affiliations:** 1 Mushroom Research Centre, Faculty of Science, University of Malaya, 50603 Kuala Lumpur, Malaysia; 2 Institute of Biological Sciences, Faculty of Science, University of Malaya, 50603 Kuala Lumpur, Malaysia

**Keywords:** 6-Shogaol, Ginger, Nerve growth factor, Neuritogenesis, PC-12 cells, NGF mimics, TrkA, MEK/ERK1/2, PI3K/AKT, Neurodegenerative disease

## Abstract

**Background:**

Ginger is a popular spice and food preservative. The rhizomes of the common ginger have been used as traditional medicine to treat various ailments. 6-Shogaol, a pungent compound isolated from the rhizomes of *jahe gajah* (*Zingiber officinale var officinale*) has shown numerous pharmacological activities, including neuroprotective and anti-neuroinflammatory activities. The aim of this study was to investigate the potential of 6-shogaol to mimic the neuritogenic activity of nerve growth factor (NGF) in rat pheochromocytoma (PC-12) cells.

**Methods:**

The cytotoxic effect of 6-shogaol was determined by 3-(4,5-dimethythiazol-2-yl)-2,5-diphenyltetrazolium bromide (MTT) assay. The neuritogenic activity was assessed by neurite outgrowth stimulation assay while the concentration of extracellular NGF in cell culture supernatant was assessed by enzyme-linked immunosorbent assay (ELISA). Involvement of cellular signaling pathways, mitogen-activated protein kinase kinase/extracellular signal-regulated kinase1/2 (MEK/ERK1/2) and phosphoinositide-3-kinase/protein kinase B (PI3K/AKT) in 6-shogaol-stimulated neuritogenesis were examined by using specific pharmacological inhibitors.

**Results:**

6-Shogaol (500 ng/ml) induced neuritogenesis that was comparable to NGF (50 ng/ml) and was not cytotoxic towards PC-12 cells. 6-Shogaol induced low level of NGF biosynthesis in PC-12 cells, showing that 6-shogaol stimulated neuritogenesis possibly by inducing NGF biosynthesis, and also acting as a substitute for NGF (NGF mimic) in PC-12 cells. The inhibitors of Trk receptor (K252a), MEK/ERK1/2 (U0126 and PD98059) and PI3K/AKT (LY294002) attenuated the neuritogenic activity of both NGF and 6-shogaol, respectively.

**Conclusions:**

The present findings demonstrated that 6-shogaol induced neuritogenic activity in PC-12 cells via the activation MEK/ERK1/2 and PI3K/AKT signaling pathways. This study suggests that 6-shogaol could act as an NGF mimic, which may be beneficial for preventive and therapeutic uses in neurodegenerative diseases.

## Background

In recent years, members of the Zingiberaceae family have attracted enormous interest among researchers due to their popularity as spices and food preservatives, and their use in traditional medicine. Zingiberaceae is the largest family among the eight families in the order Zingiberales, with 53 genera and 1300 species that are predominantly found in tropical Asia [1–3]. One of the most well-known members of the Zingiberaceae family is the common ginger, *Zingiber officinale Roscoe var. officinale* which is a monocotyledon belonging to the subfamily Zingiberoideae [1]. Common ginger, originally from South-East Asia, was introduced to many parts of the world and has been cultivated for the past thousands of years for use as spices and traditional remedies [4, 5]. The rhizomes of common ginger are usually consumed in the fresh form; as dried powder, candy, and flavouring in tea or slices preserved in syrup [5]. The rhizomes of the common ginger have been used for a wide array of ailments conditions such as colds, nauseas, headaches, hypertension, dementia, indigestion, vomiting, fever, gastrointestinal discomfort, constipation, arthritis, and rheumatism as early as 2500 years ago [5–8].

**Fig. 1:**
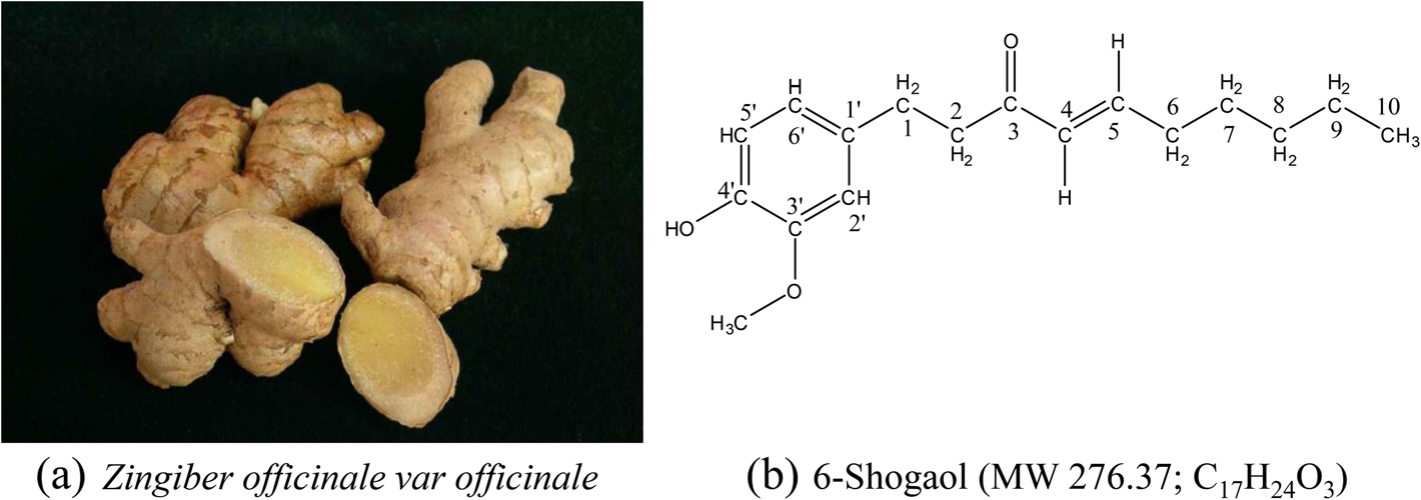
**a** Rhizome of *jahe gajah*. **b** Structure and molecular weight of 6-shogaol

There have been many scientific reports on common gingers, however chemical and biological investigations on *jahe gajah* (*Zingiber officinale var. officinale*) (Fig. 1a) are sadly lacking. ‘Jahe’ is the Indonesian word for ginger. The rhizomes of *jahe gajah* are easily interchangeable with common ginger because they are morphologically alike except for the slightly bigger size of *jahe gajah* rhizomes.

Many shogaol compounds are formed by thermal dehydration of gingerol compounds, with 6-shogaol being the most abundant [9]. 6-Shogaol is a promising compound with a number of pharmacological activities, including antioxidant [10], anti-inflammatory [10, 11], anti-neuroinflammatory [12], anti-cathartic [13], anti-neoplastic [14], and hypotensive effects [15, 16]. A number of studies documented the neuroprotective activity of 6-shogaol in vitro and in vivo. Kim and Kim (2004) [17] reported the neuroprotective effect of ten synthesized shogaol compounds, including 6-shogaol, in the protection of the rat pheochromocytoma (PC-12) and human neuroblastoma (IMR-32) cells from the β-amyloid insult. Kyung et al. (2006) [18] found that rats with spinal cord injury showed recovery of hind limb reflexes more rapidly when treated with 6-shogaol. Shim and Kwon (2012) [19] reported that 6-shogaol protected cholinergic neurons from reactive oxygen species (ROS) in hippocampal neuronal (HT22) cells. Study by Moon et al. (2014) [20] revealed that administration of 6-shogaol significantly enhanced learning and memory in both memory-impaired and normal mice. A recent study by Peng et al. (2015) [21] reported that 6-shogaol possessed anti-oxidant and cytoprotective effects against oxidative stress-induced cell damage in PC-12 cells. Peng et al. (2015) [21] further suggested that 6-shogaol may be a potential candidate for the prevention of oxidative stress-mediated neurodegenerative disorders. However, to date, there are limited reports on the neuritogenic activity of the chemical compounds isolated from common ginger, notably 6-shogaol.

Neuritogenesis is an important and complex process in the brain development, associated with neuronal differentiation, sprouting and extensions of neurites to form a functional and communications network within the neuronal cells [22]. Neurotrophins are the key regulators of neuritogenesis. Neurotrophins such as NGF are mandatory for the development and maintenance of the sympathetic and parasympathetic nervous system [23–25]. Nerve growth factor is produced in the neocortex and the hippocampus [26], to maintain the cholinergic neurons of the forebrain [25, 27]. Deprivation of NGF may affect the cholinergic neurons, causing neuronal atrophy, memory impairments, and further leads to neurodegenerative diseases, including Alzheimer’s diseases [28–31]. However, therapeutic application of NGF is restricted by its high molecular weight polypeptide structure. Nerve growth factor is composed of two non-covalently bonded polypeptide chains where each chain consists of 118 amino acids (with total molecular weight of 29,000) [32, 33]. Peripheral administration of NGF does not significantly penetrate the blood-brain barrier (BBB) and blood-nerve barrier (BNB) [34, 35]. Therefore, low molecular weight NGF mimics from natural or dietary sources have become the center of attention in the search for preventive and therapeutic agents of neurodegenerative diseases. 6-Shogaol is a phenolic compound with an alkyl side-chain consisting of an α, β-unsaturated ketone moiety (Fig. 1b). It is also a small molecule with molecular weight of 276.37. Thus 6-shogaol may penetrate the BBB and BNB than NGF.

Cultured PC-12 cell line, a clonal population arising from a tumor of the rat adrenal medulla, has been used extensively as model for the study of the actions of NGF, neuronal differentiations and neuronal signaling pathways [36, 37]. In the presence of NGF, PC-12 cells undergo a profound and easily observable neuritogenesis [36, 37]. Addition of nanomolar amounts of NGF to PC-12 cells leads to mitotic arrest, differentiation into a sympathetic-like neuronal phenotype, and an increase the expression of neuronal proteins [38]. The aims of the present study were to investigate the NGF-mimicking activities of 6-shogaol by exploring the neuritogenic activity of 6-shogaol, as well as the involvement of NGF responsive signaling pathways of 6-shogaol-induced neuritogenesis in PC-12 cells.

## Methods

### Materials and chemicals

The rat pheochromocytoma (PC-12) cell line was purchased from ATCC (American Type Culture Collection). Kaighn’s Modification of Ham’s F-12 Medium (F-12 K medium), NGF-7S from murine submaxillary gland, 3-(4,5-dimethythiazol-2-yl)-2,5-diphenyltetrazolium bromide (MTT), phosphate buffered saline (PBS), dimethyl sulfoxide (DMSO), tropomyosin receptor kinase (Trk) receptor inhibitor (K252a), mitogen-activated protein kinase kinase/extracellular signal-regulated kinase (MEK/ERK1/2) inhibitors (U0126, PD98059), phosphoinositide-3-kinase/protein kinase B (PI3K/AKT) inhibitor (LY294002), were purchased from Sigma Co. (St. Louis, MO, USA). Fetal bovine serum (FBS) and horse serum (HS) were purchased from PAA Laboratories (Cölbe, Germany). ChemiKine™ nerve growth factor sandwich enzyme-linked immunosorbent assay (ELISA) kit was purchased from Chemicon® International, Inc. (USA). All chemicals used were of analytical grade.

### Plant material

The rhizome of *jahe gajah*, a variant of common ginger (*Zingiber officinale* var. *officinale*), was obtained from Yogyakarta, Indonesia in July 2006. The rhizome of *jahe gajah* was authenticated by Professor Dr. Halijah Ibrahim. A voucher specimen (voucher number: HI1364) was deposited in the Herbarium of the Institute of Biological Sciences, Faculty of Science, University of Malaya, 50,603 Kuala Lumpur, Malaysia.

### Isolation of 6-shogaol from ethyl acetate extract of *jahe gajah*

The extraction and fractionation of the rhizomes of *jahe gajah* was conducted as described in Malek et al. (2011) [39]. Briefly, 1.0 kg of the ground powdered sample of the rhizomes of *jahe gajah* was extracted with methanol and the extracting solvent was evaporated using a rotary evaporator to obtain the crude extract. The crude extract was then successively fractionated with hexane, ethyl acetate and water to yield hexane (JHG), ethyl acetate (JEG) and water fractions (JWG). The JEG was subjected to vacuum liquid chromatography for further fractionation and eleven sub-fractions (JEGF1 to JEGF11) were obtained. 6-Shogaol was isolated from the sub-fraction JEGF5 via semi-preparative high performance liquid chromatography using a slightly modified method described by Jolad et al. (2005) [9] on a Shimadzu LC system equipped with a Shimadzu LC-10AT VP pump, Shimadzu SCL-10A VP system controller, Shimadzu SPD-M10A VP Photo 4544 Diode Array detector, Shimadzu DGU-12A vacuum degasser and Shimadzu LC Solution software. The solvents used for High-performance liquid chromatography (HPLC) were of chromatographic grade acetonitrile (J.T. Baker), methanol (J.T. Baker) and ultra-pure water (H_2_O). The column used was a Chromolith Performance RP-18e (100.0 mm X 10.0 mm i.d.) for preparative scale separation. Identification of 6-shogaol, an yellowish oil with light odour of pungency, was determined using spectrometry and spectroscopy techniques and comparison of the obtained data with those from the literature [9, 40–42].

### In vitro cell culture

The PC-12 cells in complete F-12 K medium supplemented with 15% (*v*/v) of heat-inactivated HS and 2.5% (*v*/v) of heat-inactivated FBS were maintained at 37 °C in a 5% CO_2_-humidified incubator. The cells upon reaching 80% confluent were passaged every three days.

### Assessment of cytotoxicity of 6-shogaol towards PC-12 cells

Cells at a density of 1 X 10^4^ cells per well were plated in 96-well plates and incubated overnight at 37 °C in a 5% CO_2_-humidified incubator. After 24 h of incubation, the supernatant was carefully replaced with freshly prepared 6-shogaol (0–1250 μg/ml) in complete F-12 K medium. After 48 h of incubation, 20 μl of MTT (5 mg/ml) was added into each well and incubated at 37 °C for 4 h. Subsequently, the supernatant was discarded and 100 μl of DMSO was added into each well to dissolve the MTT formazan crystals, mixed thoroughly and incubated for 15 min. The extent of the reduction of MTT was determined by measurement of the absorbance at 540 nm with 690 nm as background absorbance with an ELISA microplate reader (Sunrise, Tecan, Austria). The cells incubated in the medium only were denoted as the negative control while the complete F-12 K medium was the blank. The 50% inhibitory concentration (IC_50_) was interpolated from the response curve.

### Assessment of neuritogenic activity of 6-Shogaol in PC-12 cells

#### Neurite outgrowth stimulation assay

Cells at a density of 5 X 10^3^ cells per well were seeded in 12-well plates and then treated with freshly prepared 6-Shogaol (0 to 10,000 ng/ml) in complete F-12 K medium. Cells in complete F-12 K medium without treatment served as a negative control while cells treated with 50 ng/ml of NGF served as a positive control. Assay plates were incubated for 48 h at 37 °C in a 5% CO_2_-humidified incubator prior to quantification of the neurite-bearing cells.

#### Quantification of neurite bearing-cells

Differentiated cells were counted by visual examination of the microscopic field. A neurite-bearing cell was defined as a cell with one or more axon-like extension that was double or more the length of the cell body diameter [43]. Ten selected microscopic fields with an average of 200–300 cells per well were assessed under an inverted microscope (Nikon Eclipse TS100). The images were captured with a QImaging Go-3 color CMOS Camera (QImaging, Canada) and by the image processor system, Image-Pro Insight (MediaCybernetics, MD). The percentage of neurite-bearing cells was evaluated by scoring the proportion of neurite-bearing cells to the total number of cells in a well.

#### Immunocytofluorescence staining of neurofilaments

The PC-12 cells were stained by anti-neurofilament 200 antibody and fluorophore-conjugated secondary antibody as previously described [44]. The slides were observed under fluorescence illumination using fluorescein isothiocyanate (FITC) and 4–6-Diamidino-2-phenylindole (DAPI) filters and images were captured with Nikon’s Imaging Software, NIS-Elements.

### Assessment of the concentration of extracellular NGF in cell culture supernatant

Cells at a density of 1 X 10^4^ cells per well were plated in 96-well plates. The cells were treated with freshly prepared 6-shogaol (50 to 1000 ng/ml) in complete F-12 K medium for 48 h. The cell culture supernatant was collected, centrifuged at 1500×g for 15 min and maintained at 0–4 °C prior to assay. The samples were diluted with sample diluent at a ratio of 1:2 (*v*/v). The amount of NGF in culture supernatant was measured by using ChemiKine™ nerve growth factor sandwich ELISA kit according to the manufacturer’s protocol.

### Treatment with specific inhibitors of signaling pathways

The Trk receptor inhibitor (K252a), MEK/ERK1/2 inhibitors (U0126, PD98059) and PI3K/AKT inhibitor (LY294002) were used in this study. Stock solutions (10 mM) of inhibitors were prepared in DMSO and stored at −20 °C in the dark. Final concentrations of 100 nM of K252a, 10 μM of U0126, 30 μM of LY294002 and 40 μM of PD98059 were freshly prepared by diluting in complete F-12 K medium before use. Cells were pre-incubated either with or without the inhibitor for 1 h at 37 °C in a 5% CO_2_-humidified incubator prior to the treatment of 50 ng/ml (*w*/*v*) of NGF or 500 ng/ml (*w*/*v*) of 6-Shogaol (the optimum concentration for the induction of neurite outgrowth). Cells were then incubated for 48 h prior to scoring the neurite-bearing cells.

### Statistical analysis

All the experimental data were expressed as the mean ± standard deviation (SD) of triplicate values. Statistical differences between groups were assessed using one-way analysis of variance (ANOVA) of a minimum of three independent experiments and Duncan’s multiple range test (DMRT), *p* < 0.05 was considered to be significant.

## Results

### Isolation of 6-shogaol

**Fig. 2:**
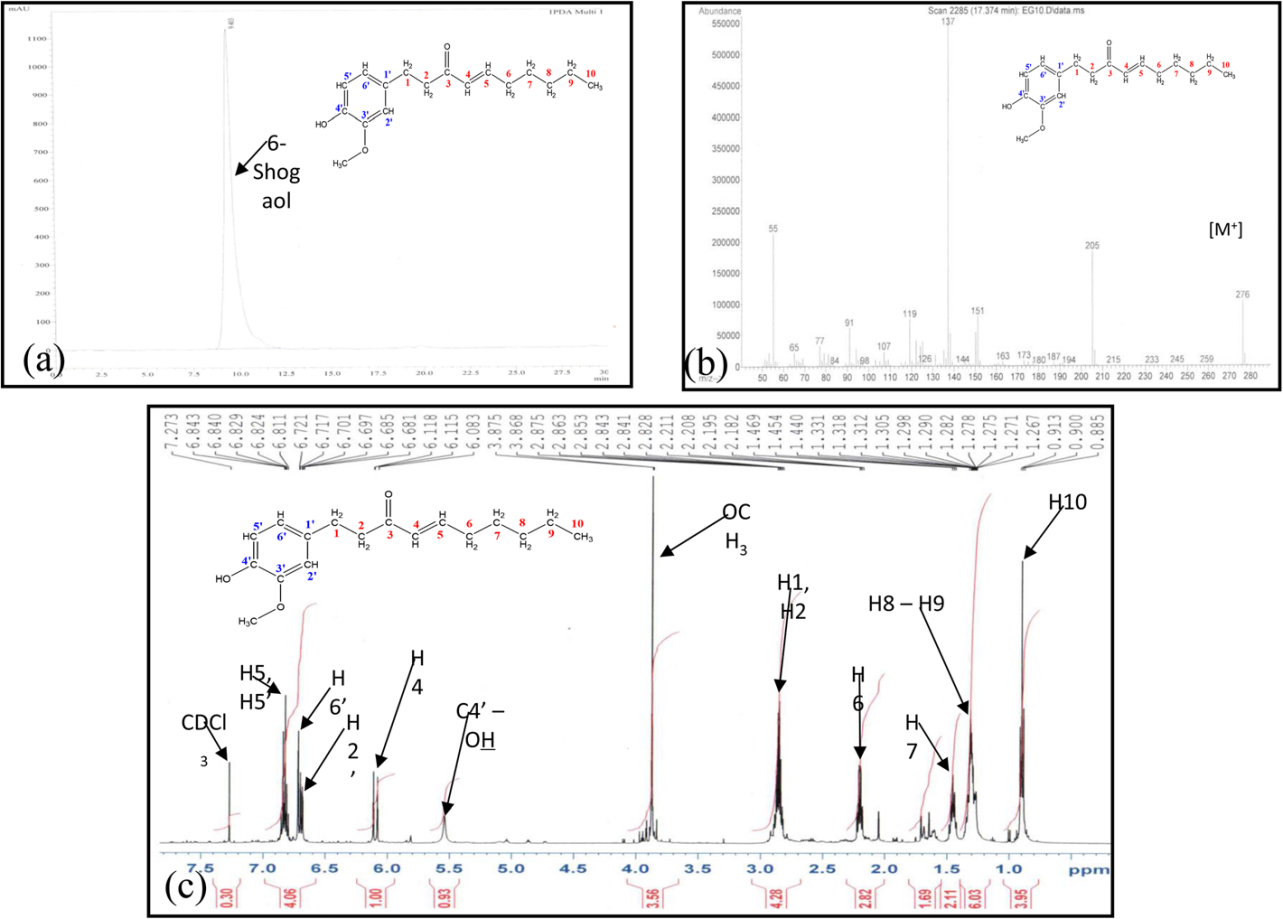
**a** HPLC chromatogram, (**b**) mass spectrum, and (**c**) proton nuclear magnetic resonance (NMR) spectrum of the purified 6-shogaol

The extraction and fractionation of 1.0 kg of ground powdered sample of the rhizomes of *jahe gajah* yielded 30.4 g of ethyl acetate fraction (JEG). JEG (3.0 g) was fractionated into eleven sub-fractions (JEGF1 to JEGF11) using vacuum liquid chromatography. Sub-fraction JEGF5 was subjected to further separation using semi-preparative high performance liquid chromatography and yielded 48.0 mg of 6-shogaol. The purified 6-shogaol (Fig. 1b) (PubChem CID: 5,281,794) [IUPACL: (E)-1-(4-hydroxy-3-methoxyphenyl)dec-4-en-3-one] was a yellowish oil with light pungent odour. It was identified using spectrometry and spectroscopy techniques and comparison of the data in the literature [9, 40–42]. The HPLC chromatogram, mass spectrum and proton nuclear magnetic resonance (NMR) spectrum of the purified 6-shogaol was shown in Fig. 2.

### Cytotoxic effect of 6-shogaol on PC-12 cells

**Fig. 3:**
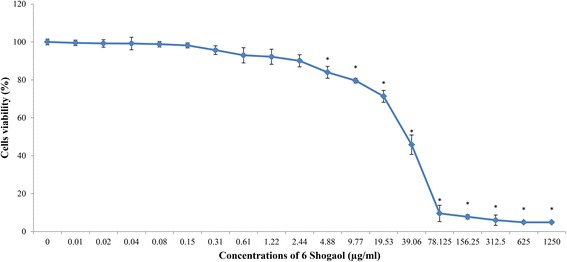
Cytotoxic effect of 6-shogaol on PC-12 cells. Cells were incubated with 6-shogaol at increasing concentrations up to 1250 μg/ml for 48 h. The mean absorbance obtained using complete F-12 K medium with cells only was designated as 100%. Data were expressed as means ± SD from three independent experiments carried out in triplicates. *p < 0.05 compared to the respective control

The cytotoxic effect of 6-shogaol on PC-12 cells after 48 h was determined. The responses of PC-12 cells towards increasing concentrations of 6-shogaol is shown in Fig. 3. The viability of cells decreased in a dose-dependent manner. The percentage of viable cells decreased significantly (*p* < 0.05) starting at 4.88 μg/ml (87.88 ± 0.93%) of 6-shogaol. The 50% inhibitory concentration (IC_50_) values of 6-shogaol after 48 h treatment was 20.04 μg/ml (72.51 μM). The concentrations of 6-shogaol used for the following assays were at 0 to 10,000 ng/ml, ensuring that the percentage of viable cells maintained at 90% and above.

### Neuritogenic effect of 6-shogaol in PC-12 cells

**Fig. 4:**
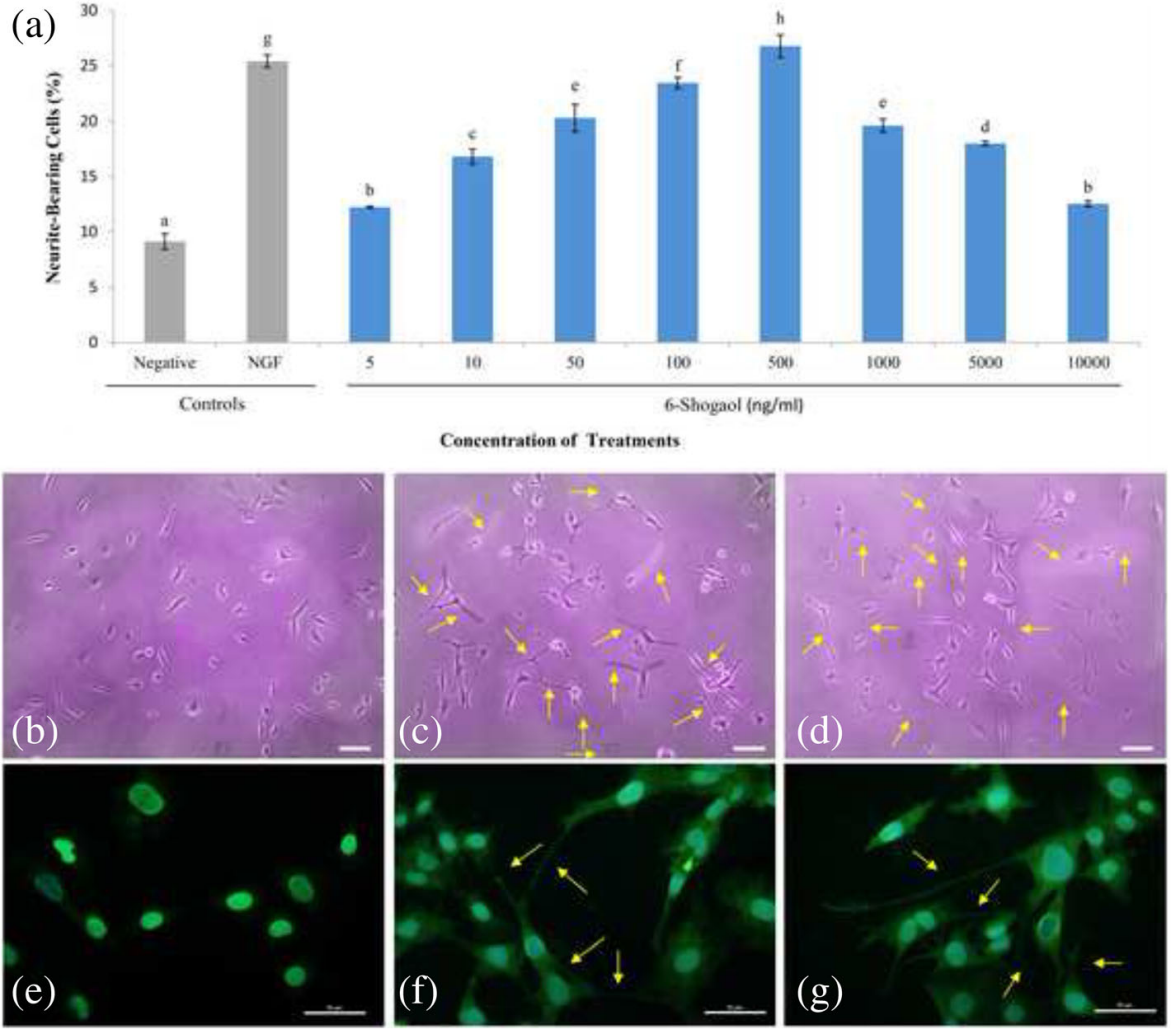
Neuritogenic effect of 6-shogaol in PC-12 cells. **a** Percentage of neurite-bearing cells after 48 h of incubation with NGF (50 ng/ml) (positive control) or 6-shogaol (5 to 10,000 ng/ml). Cells in complete F-12 K medium served as a negative control. Data were expressed as means ± SD from three independent experiments carried out in triplicates. Means with different alphabets show significant differences (*p* < 0.05). Phase contrast photomicrographs of PC-12 cells: (**b**) in medium only; (**c**) treated with 50 ng/ml of NGF; (**d**) treated with 500 ng/ml of 6-shogaol. Scale bar = 20 μm. Immunocytofluorescence staining of neurofilaments of PC-12 cells: (**e**) in medium only; (**f**) treated with 50 ng/ml of NGF; (**g**) treated with 500 ng/ml of 6-shogaol. Scale bar = 50 μm. Arrows indicate neurite extensions

PC-12 cells require NGF to induce neuritogenesis. Nerve growth factor-induced neuritogenesis in PC-12 cells served as a control to compare the neuritogenic effect of 6-shogaol. Treatment of NGF (50 ng/ml) and 6-shogaol (5–10,000 ng/ml) significantly (*p* < 0.05) induced neuritogenesis in PC-12 cells after 48 h of incubation (Fig. 4a). 6-Shogaol increased the percentage of neurite bearing cells in a concentration-dependent manner at lower concentrations (5–500 ng/ml). In contrast, when the concentrations of 6-shogaol were increased gradually (1000–10,000 ng/ml), the percentage of neurite bearing cells decreased gradually. Nerve growth factor increased the percentage of neurite bearing cells (25.40 ± 0.57%) by approximately 2.8-fold in comparison to the negative control (9.11 ± 0.71%). 6-Shogaol induced maximal neurite bearing cells (26.88 ± 1.02%) at 500 ng/ml, which is comparable to the NGF. Figure 4 b-d shows the morphological changes of PC-12 cells of different treatments after 48 h. In the negative control, the cells are relatively rounded with few visible neurites (Fig. 4b). The cells were elongated with significant neurite extensions that were double the length of cell body diameter in 50 ng/ml of NGF- (Fig. 4c), and 500 ng/ml of 6-shogaol- (Fig. 4d) treated cells. Immunostaining of neurofilaments confirmed the neurite extensions were stimulated by NGF (Fig. 4f) and 6-shogaol (Fig. 4g). PC-12 cells nuclei were stained blue by DAPI and neurofilaments were stained green by anti-NF-200 antibody labelled with FITC.

### Induction of NGF biosynthesis by 6-shogaol in PC-12 cells

**Fig. 5:**
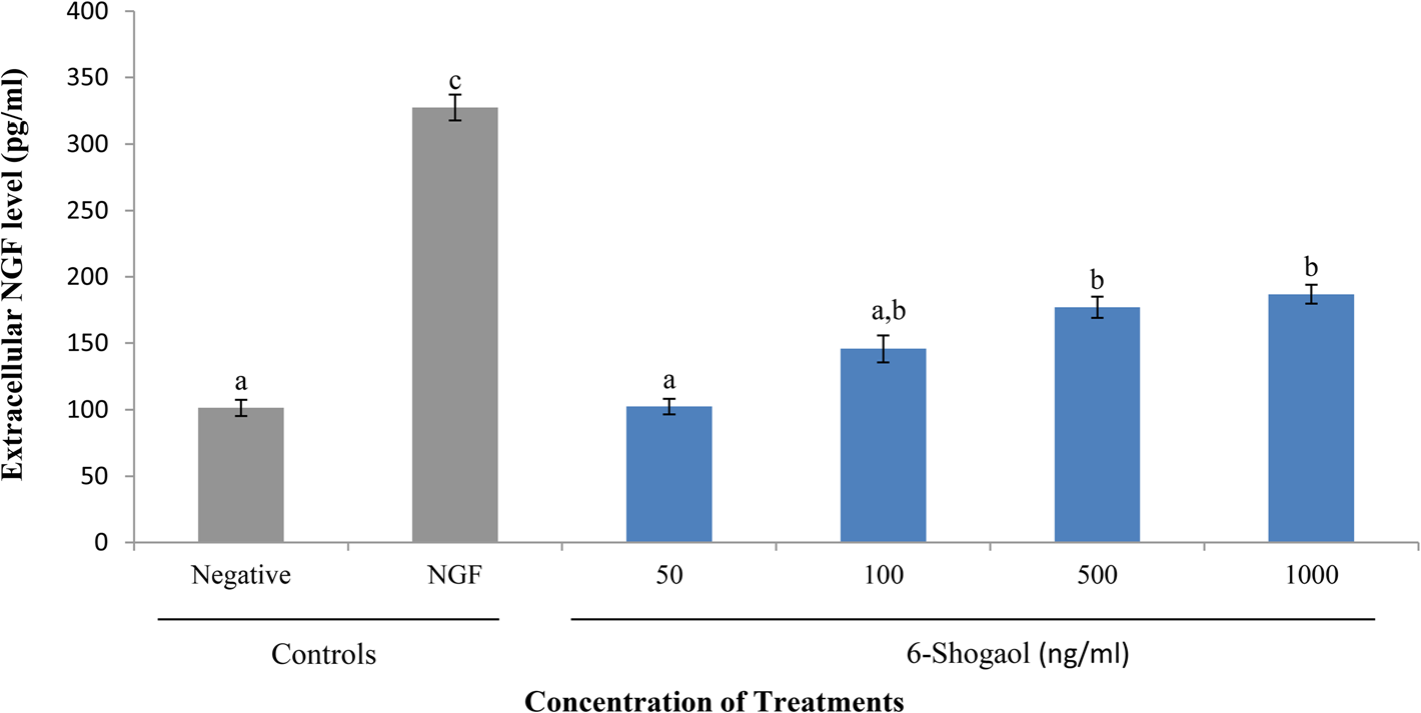
Extracellular NGF concentration in supernatants of NGF- and 6-shogaol-treated PC-12 cells. Cells were incubated with NGF (50 ng/ml) or 6-shogaol for 48 h. Cells in complete F-12 K medium served as a negative control. The concentration of extracellular NGF (pg/ml) was interpolated from the NGF standard curve using the average absorbance value of triplicate wells. Data were expressed as means ± SD from three independent experiments carried out in triplicates. Means with different alphabets show significant difference (*p* < 0.05)

To determine the potential of 6-shogaol in inducing biosynthesis of NGF in PC-12 cells, the concentration of NGF in cell supernatants after 48 h of incubation was assessed. The extracellular concentration of NGF in the positive control, 50 ng/ml of NGF (327.37 ± 9.76 pg/ml) was significantly (*p* < 0.05) higher by approximately 3.2-fold, in comparison to the negative control (101.51 ± 6.15 pg/ml) (Fig. 5). The extracellular concentrations of NGF in 6-shogaol-treated cells increased in a concentration-dependent manner. There was no significant difference (*p* > 0.05) between the extracellular concentration of NGF in both the negative control and 6-shogaol-treated cells at 50 ng/ml. Higher concentrations of 6-shogaol promoted higher levels of NGF biosynthesis in PC-12 cells. The extracellular concentrations of NGF in cells treated with 500 and 1000 ng/ml of 6-shogaol was 177.12 and 186.88 pg/ml, respectively. Although these extracellular concentration of NGF are similar, the neuritogenic effect of 500 ng/ml 6-shogaol was significantly higher than 1000 ng/ml (Fig. 5). This may due to the increasing cytotoxicity of 1000 ng/ml 6-shogaol.

### The involvement of signaling pathways in 6-shogaol-induced neuritogenesis

**Fig. 6:**
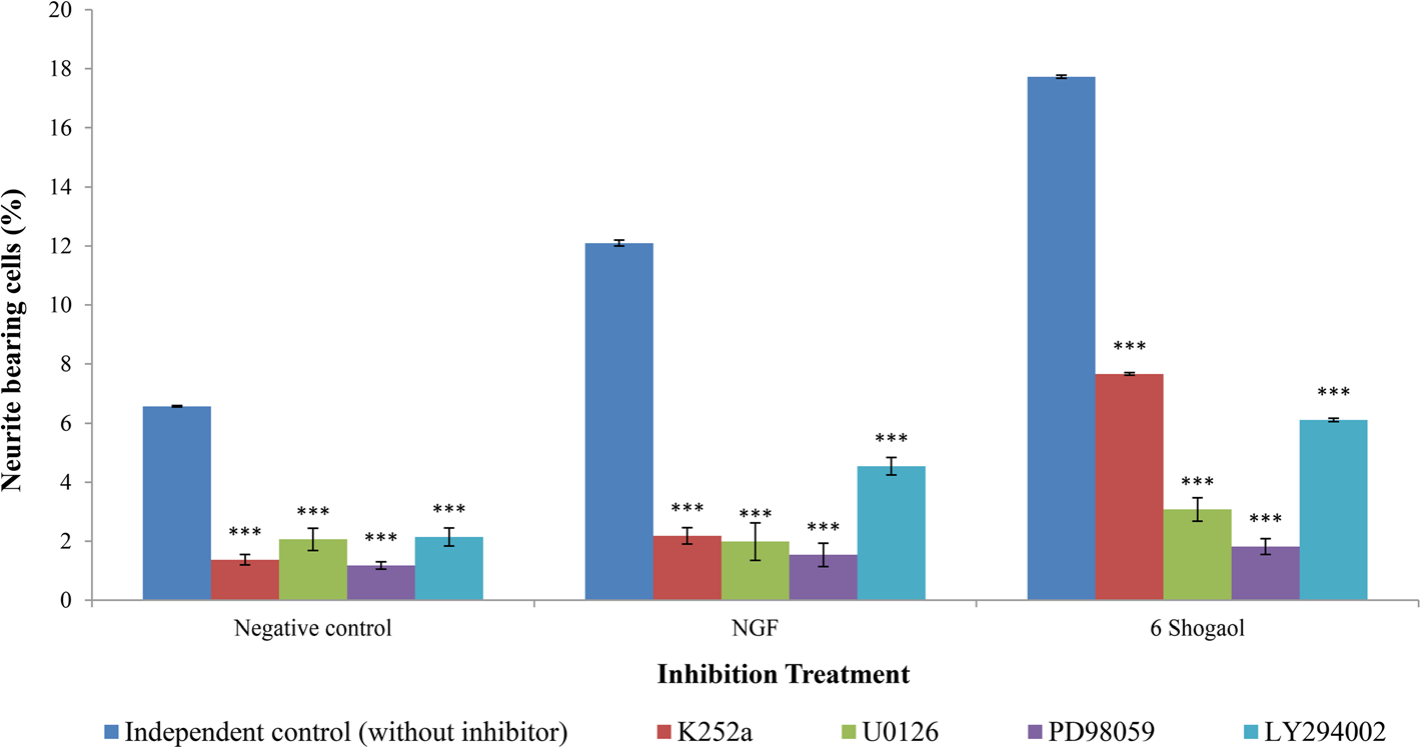
Inhibition effect of the specific inhibitors on NGF-, or 6-shogaol induced neuritogenesis in PC-12 cells. Cells were pre-treated with K252a, U0126, PD98059 and LY294002 for one hour before the treatment with NGF (50 ng/ml) or 6-shogaol (500 ng/ml). Cells in complete F-12 K medium served as a negative control. An independent control (without inhibitor) was used in each treatment group. Data were expressed as means ± SD from three independent experiments carried out in triplicates. ***p < 0.001 compared to the respective controls

The involvement of NGF high affinity receptor (TrkA) and NGF responsive signaling pathways (MEK/ERK1/2 and PI3K/AKT) in 6-shogaol-treated PC12 cells were assessed by using Trk inhibitor (K252a), MEK1/2 inhibitors (U0126 and PD98059) and PI3K inhibitor (LY294002), respectively. Cells treated with only NGF or 6-shogaol without inhibitors (independent control) induced neuritogenesis in PC-12 significantly (*p* < 0.001) compared to cells treated with inhibitors (Fig. 6). Pre-treatment with Trk, MEK1/2 and PI3K inhibitors attenuated the neuritogenic activity of both NGF- and 6-shogaol-induced neuritogenesis significantly (*p* < 0.001). Trk inhibitor, K252a decreased the percentage of neurite bearing cells of NGF- and 6-shogaol-treated cells by approximately 81.94% and 56.78%, respectively. MEK1/2 inhibitors, U0126 and PD98059 decreased the percentage of neurite bearing cells of NGF- and 6-shogaol-treated cells by approximately 83.55% and 82.62% (U0126), and 87.27% and 89.73% (PD98059), respectively. Meanwhile, the PI3K inhibitor, LY294002 decreased the percentage of neurite bearing cells of NGF- and 6-shogaol-treated cells by approximately 62.40% and 65.47%, respectively.

## Discussion

The cytotoxicity assay is a crucial preliminary analysis to identify the safety doses of 6-shogaol. An in vivo acute toxicity study by Suekawa et al., 1984 [16] demonstrated that the 6-shogaol showed lower toxicity (about one half) compared to that of 6-gingerol in all the administration routes, including oral (p.o), interperitoneal (i.p) and intravenous (i.v). Cell culture models are often used to investigate the in vitro toxicity of compounds. A recent study by Zhu et al. (2013) [45] disclosed that 6-shogaol exhibited low toxicity in normal cells whereby the IC_50_ of 6-shogaol on normal colon (CCD-18Co) and lung (IMR-90) cell lines after 24 h of incubation were 43.91 μM (12.14 μg/ml) and 36.65 μM (10.13 μg/ml), respectively. In the present study, the IC_50_ values of 6-shogaol on PC-12 cells viability after 48 h treatment was 20.04 μg/ml (72.51 μM). Overall, low concentrations of 6-shogaol was non-cytotoxic towards both in vitro and in vivo models.

The present study demonstrated the potential of 6-shogaol as potent neuritogenic compound. 6-Shogaol at 500 ng/ml successfully induced approximately 3-fold higher of neurite bearing cells (26.88 ± 1.02%) compared to the negative control (9.11 ± 0.71%) and was comparable to that of NGF (25.40 ± 0.57%). A recent study by Kubo (2015) [46] found several new neuritogenic compounds isolated from Indonesian ginger *Zingiber purpureum*, including phenylbutenoid dimers 3–4 and curcuminoids 5–6 that significantly induced neuritogenesis in PC-12 cells and increased the neurite length and neurite bearing cells in primary rat cortical neurons. Curcumin, a compound isolated from turmeric, *Curcuma longa* Linn. (Zingiberaceae) was also found to possess potent neuritogenic activity in PC-12 cells [47, 48]. Liao et al. (2012) [47] reported that curcumin induced maximal percentage of neurite bearing cells (21.6 ± 2.0%) at 20 μM (5.53 μg/ml), but, was lower compared to 50 ng/ml NGF (23.3 ± 1.9%). John et al. (2013) [48] found that curcumin induced maximal percentage of neurite bearing cells of 29.5% at 10 μg/ml, and this result was significantly higher in comparison to that by 50 ng/ml of NGF (21.45%). Consistent with the reports by Liao et al. (2012) [47] and John et al. (2013) [48], the present study showed the ability of 6-shogaol to independently induce neuritogenesis which was comparable to the NGF in PC-12 cells. Well-known neuroactive compounds, such as hericinones C, D and E isolated from a very popular edible and medicinal mushroom, *Hericium erinaceus* (Bull.:Fr) Pers.*,* were not able to trigger neuritogenesis when they were used alone [49]. Phan et al. (2014) [49] showed that these compounds required a combination treatment with low concentration of NGF to enhance the neuritogenesis in PC-12 cells. The present study revealed that 6-shogaol is a potential neuroactive compound which mimics the neuritogenic activity of NGF independently (in vitro).

Several natural compounds of low molecular weight were reported to stimulate NGF biosynthesis in vitro, including propentofylline [50], 1,4-benzoquinone [51], hericenones C-H [49, 52, 53], erinacine A-G [54–56], and erinacine H-I [57]. According to Phan et al. (2014) [49], hericenone E alone did not induce neuritogenesis nor NGF biosynthesis in PC-12 cells. Hericenone E with addition of low concentration of NGF (5 ng/ml) successfully induced almost double of NGF biosynthesis in PC-12 cells compared to the positive control, 50 ng/ml NGF alone [49]. In the present study, 500 ng/ml of 6-shogaol induced only low level of NGF biosynthesis in PC-12 cells. This indicated that 6-shogaol induced neuritogenesis independently in PC-12 by inducing low level of NGF biosynthesis and acting as a substitute for NGF (NGF mimic) in PC-12 cells.

**Fig. 7:**
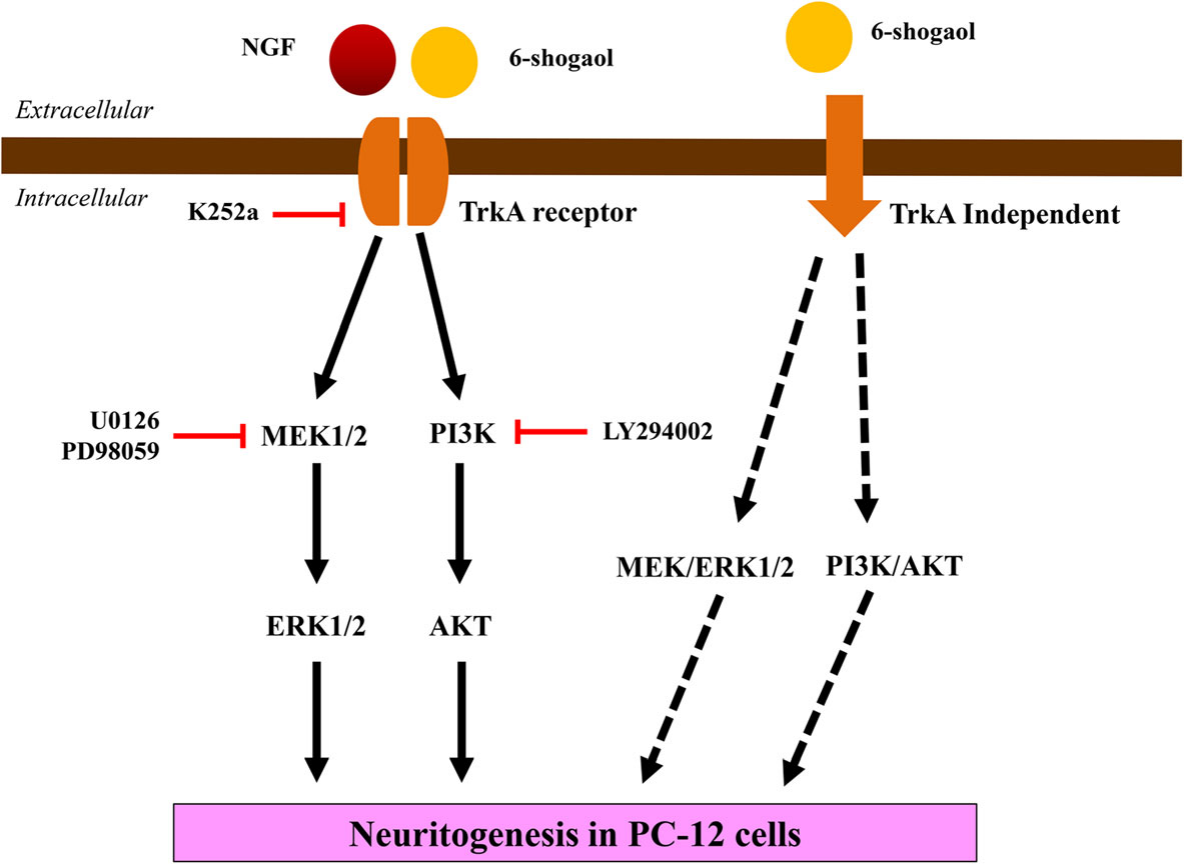
Hypothetical mechanisms of NGF and 6-shogaol neuritogenesis in PC-12 cells**.** Nerve growth factor induced neuritogenesis via binding to its high affinity TrkA receptor and leads to the activation of two major signaling pathways, the MEK/ERK1/2 and PI3K/AKT. Addition of Trk inhibitor (K252a), MEK/ERK1/2 inhibitors (U0126, PD98059) and PI3K/AKT inhibitor (LY294002) inhibited NGF-induced neuritogenesis in PC-12 cells. In the present study, the neuritogenic activity of 6-shogaol was inhibited by U0126, PD98059 and LY294002, but only partially inhibited by K252a. Therefore, based on the present findings, both the TrkA-dependent and TrkA-independent initiated MEK/ERK1/2 and PI3K/AKT signaling pathways were involved in 6-shogaol-induced neuritogenesis in PC-12 cells

Treatment of NGF in PC-12 cells is associated with the expression of TrkA receptor and the initiation of two predominant signaling pathways, the MAPK/MEK/ERK and PI3K/AKT pathways, which eventually lead to neuritogenesis [58, 59]. Biological actions of NGF are mediated by its high-affinity TrkA receptor [60]. K252a acts as a potent inhibitor of Trk neurotrophin receptor proteins [61]. It has been shown that K252a selectively inhibits the actions of NGF in PC-12 cells [62]. In the present study, K252a inhibited the neuritogenic activity of NGF in PC-12 cells by approximately 82%, compared to the independent control (cells treated with only NGF without inhibitor). However, the neuritogenic activity of 6-shogaol was only partially (56.78%) inhibited by K252a, compared to the independent control (cells treated with only 6-shogaol without inhibitor). This data suggesting that the NGF-mimicking neuritogenic effects of 6-shogaol was not merely mediated through the TrkA receptor. This is consistent with the study by Phan et al. (2014) [49], showed that hericenone E-potentiated neuritogenesis is also partially (46%) inhibited by K252a. In this communication, Phan et al. (2014) [49] indicated that NGF-induced neuritogenesis potentiated by hericenone E was not entirely TrkA-dependant in PC-12 cells. In the present study, the results indicated that there may be other neurotrophin receptor(s) and/or TrkA-independent signaling pathway(s) involved in 6-shogaol-induced neuritogenic activity in PC-12 cells (Fig. 7). Further investigation on the interaction between the 6-shogaol and other neurotrophin receptors and their related signaling pathways in 6-shogaol-induced neuritogenic activity is warranted.

Persistent phosphorylation and activation of MEK/ERK1/2 and PI3K/AKT signaling pathways are associated with several cellular processes including proliferation, survival, neuritogenesis, and regeneration [63, 64]. Priming PC-12 cells with MEK1/2 inhibitors (U0126 and PD98059) and PI3K inhibitor (LY294002) leads to inhibition of both the phosphorylation and activation of MEK/ERK1/2 and PI3K/AKT, respectively, and eventually diminishes the interrelated cellular processes [65–67]. Liao et al. (2012) [47] demonstrated that curcumin-induced neuritogenesis was mediated by MEK/ERK1/2 signaling pathway in PC-12 cells. Addition of U0126 significantly abolished the curcumin-induced neuritogenesis in PC-12 cells; the percentage of neurite bearing cells was reduced from 21.6 ± 2.4% to 4.1 ± 1.6%, moreover, the phosphorylation of ERK1/2 (Thr202/Tyr204) was also inhibited [47]. A study by El Omri et al. (2012) [68] showed that U0126 and LY294002 attenuated luteolin- (a neuroactive compound isolated from *Rosmarinus officinalis*) induced acetylcholinesterase (neuronal differentiation marker) and neuritogenic activity in PC-12 cells. El Omri et al. (2012) [68] concluded that the activation of ERK1/2 and AKT signaling were involved in the neuritogenic and cholinergic activities of luteolin. The present findings demonstrated that 6-shogaol-induced neuritogenesis in PC12 cells was significantly attenuated by the addition of U0126, PD98059, and LY294002 inhibitors, respectively. The present findings suggest that both MEK/ERK1/2 and PI3K/AKT signaling pathways were mandatory in 6-shogaol-induced neuritogenesis.

## Conclusions

In summary, low concentrations of 6-shogaol was not cytotoxic and induced neuritogenesis in PC-12 cells. Neuritogenic effect of 6-shogaol may be due to 6-shogaol mimicking NGF activity and inducing biosynthesis of NGF in PC-12 cells. The present findings showed that 6-shogaol induced neuritogenesis through the activation of both TrkA-dependent and TrkA-independent initiated NGF responsive signaling pathways, the MEK/ERK1/2 and PI3K/AKT pathways in PC-12 cells. The present study also showed that 6-shogaol could act as an NGF mimic, which may be beneficial for preventive and therapeutic uses in neurodegenerative diseases. However, further studies are necessary to validate these activities of 6-shogaol, including the potential of BBB permeability, pharmacological activities, and mechanisms in both in vitro and in vivo studies.

## Abbreviations

AKT: Protein kinase B
BBB: blood-brain barrier
BNB: blood-nerve barrier
ELISA: enzyme-linked immunosorbent assay
ERK: Extracellular signal-regulated kinase
MAPK: Mitogen activated protein kinase
MEK: Mitogen-activated protein kinase kinase
NGF: nerve growth factor
PC-12: cells rat pheochromocytoma cells
PI3K: Phosphoinositide-3-kinase

## References

[1] Vasala PA. Ginger. In: Peter KV, editor. Handbook of herbs and spices. England: Woodland Publishing Limited; 2001. p. 195.

[2] Kress WJ, Prince LM, Williams KJ (2002). The phylogeny and a new classification of the gingers (Zingiberaceae): evidence from molecular data. Am J Bot.

[3] Ling LR, Wahab NA, Abidin NZ (2005). Cytotoxic activity of selected Zingiberaceae. Malaysian Journal of Science.

[4] Park KK, Chun KS, Lee JM, Lee SS, Surh YJ (1998). Inhibitory effect of [6]-gingerol, major pungent principle of ginger, on phorbol ester-induced inflammation, epidermal ornithine decarboxylase activity and skin tumor promotion in ICR mice. Cancer Lett.

[5] Shukla Y, Singh M (2007). Cancer preventive properties of ginger: a brief review. Food Chem Toxicol.

[6] Lee E, Surh YJ (1998). Induction of apoptosis in HL-60 by pungent vanilloids, [6]-gingerol and [6]-paradol. Cancer Lett.

[7] Surh YJ, Lee E, Lee JM (1998). Chemoprotective properties of some pungent ingredients present in red pepper and ginger. Mutat Res.

[8] Ali BH, Blunden G, Tanira MO, Nemmar A (2008). Some phytochemical, pharmacological and toxicological properties of ginger (*Zingiber officinale Roscoe*): a review of recent research. Food Chem Toxicol.

[9] Jolad DS, Lantz RC, Chen GJ, Bates RB (2005). Commercially processed dry ginger (*Zingiber officinale*): composition and effects on LPS-induced PGE_2_ production. Phytochemistry.

[10] Dugasani S, Pichika MR, Nadarajah VD, Balijepalli MK, Tandra S, Korlakunta JN (2010). Comparative antioxidant and anti-inflammatory effects of [6]-gingerol, [8]-gingerol, [10]-gingerol and [6]-shogaol. J Ethnopharmacol.

[11] Lantz RC, Chen GJ, Sarihan M, Solyom AM, Jolad SD, Timmermann BN (2007). The effect of extracts from ginger rhizome on inflammatory mediator production. Phytomedicine.

[12] Ha SK, Moon E, Ju MS, Kim DH, Ryu JH, Oh MS (2012). 6-Shogaol, a ginger product, modulates neuroinflammation: a new approach to neuroprotection. Neuropharmacology.

[13] Huang Q, Matsuda H, Sakai K, Yamahara J, Tamai Y (1990). The effect of ginger on serotonin induced hypothermia and diarrhea. Yakugaku Zasshi.

[14] Rhode J, Fogoros S, Zick S, Wahl H, Griffith KA, Huang J (2007). Ginger inhibits cell growth and modulates angiogenic factors in ovarian cancer cells. BMC Complement Altern Med.

[15] Suekawa M, Aburada M, Hosoya E (1986). Pharmacological studies on ginger II, pressor action of 6-shogaol in anesthetised rats, or hindquarters, tail and mesenteric vascular beds of rats. J. Pharmacobio-dyn..

[16] Suekawa M, Ishige A, Yuasa K, Sudo K, Aburada M, Hosoya E (1984). Pharmacological studies on ginger I, pharmacological actions of pungent constituents, 6-gingerol and 6-shogaol. J Pharmacobio-dyn.

[17] Kim DSHL, Kim JY (2004). Side-chain length is important for shogaols in protecting neuronal cells from β-amyloid insult. Bioorg Med Chem Lett.

[18] Kyung KS, Gon JH, Geun KY, Sup JJ, Suk WJ, Ho KJ (2006). 6-Shogaol, a natural product, reduces cell death and restores motor function in rat spinal cord injury. Eur J Neurosci.

[19] Shim S, Kwon J (2012). Effects of [6]-shogaol on cholinergic signaling in HT22 cells following neuronal damage induced by hydrogen peroxide. Food Chem Toxicol.

[20] Moon M, Kim HG, Choi JG, Oh H, Lee PK, Ha SK (2014). 6-Shogaol, an active constituent of ginger, attenuates neuroinflammation and cognitive deficits in animal models of dementia. Biochem Biophys Res Commun.

[21] Peng S, Yao J, Liu Y, Duan D, Zhang X, Fang J (2015). Activation of Nrf2 target enzymes conferring protection against oxidative stress in PC12 cells by ginger principal constituent 6-shogaol. Food Funct.

[22] Da Silva JS, Dotti CG (2002). Breaking the neuronal sphere: regulation of the actin cytoskeleton in neuritogenesis. Nat Rev Neurosci.

[23] Levi-Montalcini R (1966). The nerve growth factor, its mode of action on sensory and sympathetic nerve cells. Harvey Lect.

[24] Greene LA, Shooter EM (1980). The nerve growth factor: biochemistry, synthesis and mechanism of action. Annu Rev Neurosci.

[25] Furukawa S, Furukawa Y (1990). Nerve growth factor synthesis and its regulatory mechanisms: an approach to therapeutic induction of nerve growth factor synthesis. Cerebrovasc Brain Metab Rev.

[26] Schindowski K, Belarbi K, Buée L (2008). Neurotrophic factors in Alzheimer’s disease: role of axonal transport. Genes Brain Behav.

[27] Koliatsos VE, Applegate MD, Knüsel B, Junard EO, Burton LE, Mobley WC (1991). Recombinant human nerve growth factor prevents degeneration of basal forebrain cholinergic neurons in primates. Ann Neurol.

[28] Fischer W, Wictorin K, Björklund A, Williams LR, Varon S, Gage FH (1987). Amelioration of cholinergic neuron atrophy and spatial memory impairment in aged rats by nerve growth factor. Nature.

[29] Thoenen H (1995). Neurotrophins and neuronal plasticity. Science.

[30] Hefti F, Weiner WJ (1986). Nerve growth factor and Alzheimer’s disease. Ann Neurol.

[31] Capsoni S, Ugolini G, Comparini A, Ruberti F, Berardi N, Cattaneo A (2000). Alzheimer-like neurodegeneration in aged antinerve growth factor transgenic mice. Proc Natl Acad Sci U S A.

[32] Angeletti RH, Bradshaw RA (1971). Nerve growth factor from mouse submaxillary gland: amino acid sequence. Proc Natl Acad Sci.

[33] Angeletti RH, Bradshaw RA, Wade RD (1971). Subunit structure and amino acid composition of mouse submaxillary gland nerve growth factor. Biochemistry.

[34] Granholm AC, Albeck D, Bäckman C, Curtis M, Ebendal T, Friden P (1998). A non-invasive system for delivering neural growth factors across the blood-brain barrier: a review. Rev Neurosci.

[35] Poduslo JF, Curran GL (1996). Permeability at the blood-brain and blood-nerve barriers of the neurotrophic factors: NGF, CNTF, NT-3. BDNF Mol Brain Res.

[36] Greene LA, Tischler AS, Fedoroff S, Hertz L (1982). PC12 Pheochromocytoma cultures in neurobiological research. Advances in cellular neurobiology.

[37] Guroff G, Bottenstein J, Sato G (1984). PC 12 cells as a model of neuronal differentiation. Cell culture in the neurosciences.

[38] Greene LA, Tischler AS (1976). Establishment of a noradrenergic clonal line of rat adrenal pheochromocytoma cells which respond to nerve growth factor. Proc Natl Acad Sci.

[39] Malek SN, Lee GS, Hong SL, Yaacob H, Wahab NA, Faizal Weber JF (2011). Phytochemical and cytotoxic investigations of *Curcuma mangga* rhizomes. Molecules.

[40] Fleming SA, Dyer CW, Eggington J. A convenient one-step gingerol synthesis. Synth.Commun. 1000;29(11):1933–1939.

[41] Jolad DS, Lantz RC, Solyom AM, Chen GJ, Bates RB, Timmerman BN (2004). Fresh organically grown ginger (*Zingiber officinale*): composition and effects on LPS-induced PGE_2_ production. Phytochemistry.

[42] Kim JS, Lee SI, Park HW, Yang JH, Shin TY, Kim YC (2008). Cytotoxic components from the dried of *Zingiber offinale roscoe*. Arch Pharm Res.

[43] Smalheiser NR, Schwartz NB (1987). Kinetic analysis of 'rapid onset' neurite formation in NG108-15 cells reveals a dual role for substratum-bound laminin. Brain Res.

[44] Seow SLS, Eik LF, Naidu M, David P, Wong KH, Sabaratnam V (2015). *Lignosus rhinocerotis* (Cooke) Ryvarden mimics the neuritogenic activity of nerve growth factor via MEK/ERK1/2 signaling pathway in PC-12 cells. Sci Rep.

[45] Zhu Y, Warin RF, Soroka DN, Chen H, Sang S (2013). Metabolites of ginger component [6]-shogaol remain bioactive in cancer cells and have low toxicity in normal cells: chemical synthesis and biological evaluation. PLoS One.

[46] Kubo M (2015). Search of neurotrophin-mimic natural products for prevention and treatment of neurodegenerative disease. Yakugaku Zasshi.

[47] Liao KK, Wu MJ, Chen PY, Huang SW, Chiu SJ, Ho CT (2012). Curcuminoids promote neurite outgrowth in PC12 cells through MAPK/ERK- and PKC-dependent pathways. J Agric Food Chem.

[48] John PA, Wong KH, Naidu M, Sabaratnam V, David P (2013). Combination effects of curcumin and aqueous extract of *Lignosus rhinocerotis* mycelium on neurite outgrowth stimulation activity in PC-12 cells. Nat Prod Commun.

[49] Phan CW, Lee GS, Hong SL, Wong YT, Brkljača R, Urban S (2014). *Hericium erinaceus* (bull.:Fr) Pers. cultivated in tropical conditions: isolation of hericenones and demonstration of NGF-mediated neurite outgrowth in PC12 cells via MEK/ERK and PI3K-Akt signaling pathways. Food Funct.

[50] Ichizo S, Yoshiko F, Shoei F (1990). Stimulation of nerve growth factor synthesis/ secretion by propentofylline in cultured mouse astroglial cells. Biochem Pharmacol.

[51] Takeuchi R, Murase K, Furukawa Y, Furukawa S, Hayashi K (1990). Stimulation of nerve growth factor synthesis/ secretion by 1,4-benzoquinone and its derivatives in cultured mouse astroglial cells. FEBS.

[52] Kawagishi H, Ando M, Sakamoto H, Yoshida S, Ojima F, Ishiguro Y (1991). Hericenones C, D and E, stimulators of nerve growth factor (NGF)-synthesis, from the mushroom *Hericium erinaceum*. Tetrahedron Lett.

[53] Kawagishi H, Ando M, Shinba K, Sakamoto H, Yoshida S (1993). Chromans, Hericenones F, G and H from the mushroom *Hericium erinaceus*. Phytochemistry.

[54] Kawagishi H, Shimada A, Shirai R, Okamoto K, Ojima F, Sakamoto H (1994). Erinacines a, B and C, strong stimulators of nerve growth factor (NGF)-synthesis, from the mycelia of *Hericium erinaceum*. Tetrahedron Lett.

[55] Kawagishi H, Shimada A, Hosokawa S, Mori H, Sakamoto H, Ishiguro Y (1996). Erinacines E, F, and G, stimulators of nerve growth factor (NGF)-synthesis, from the mycelia of *Hericium erinaceum*. Tetrahedron Lett.

[56] Kawagishi H, Simada A, Shizuki K, Ojima F, Mori H, Okamoto K (1996). Erinacine D, a stimulator of NGF-synthesis, from the mycelia of *Hericium erinaceum*. Heterocycl Commun.

[57] Lee EW, Shizuki K, Hosokawa S, Suzuki M, Suganuma H, Inakuma T (2000). Two novel diterpenoids, erinacines H and I from the mycelia of *Hericium erinaceum*. Biosci Biotechnol Biochem.

[58] Vaudry D, Stork PJS, Lazarovici P, Eiden LE (2002). Signaling pathways for PC-12 cell differentiation: making the right connections. Science.

[59] Jackson TR, Blader IJ, Hammonds-Odie LP, Burga CR, Cooke F, Hawkins PT (1996). Initiation and maintenance of NGF-stimulated neurite outgrowth requires activation of a phosphoinositide 3-kinase. J Cell Sci.

[60] Patapoutian A, Reichardt LF (2001). Trk receptors: mediators of neurotrophin action. Curr Opin Neurobiol.

[61] Knusel B, Hefti F (1992). K-252 compounds: modulators of neurotrophin signal transduction. J Neurochem.

[62] Koizumi S, Contreras ML, Matsuda Y, Hama T, Lazarovici P, Guroff G (1988). K-252a: a specific inhibitor of the action of nerve growth factor on PC 12 cells. J Neurosci.

[63] Marshall CJ (1995). Specificity of receptor tyrosine kinase signaling: transient versus sustained extracellular signal-regulated kinase activation. Cell.

[64] Barrie AP, Clohessy AM, Buensuceso CS, Rogers MV, Allen JM (1997). Pituitary adenylyl cyclase-activating peptide stimulates extracellular signal-regulated kinase 1 or 2 (ERK1/2) activity in a Ras-independent, mitogen-activated protein kinase/ERK kinase 1 or 2-dependent manner in PC12 cells. J Biol Chem.

[65] Favata MF, Horiuchi KY, Manos EJ, Daulerio AJ, Stradley DA, Feeser WS (1998). Identification of a novel inhibitor of mitogen-activated protein kinase kinase. J Biol Chem.

[66] Yan CYI, Greene LA (1998). Prevention of PC12 cell death by N-acetylcysteine requires activation of the ras pathway. J Neurosci.

[67] Yang W, Luo Y, Tang R, Zhang H, Ye Y, Xiang L (2013). Neuritogenic monoglyceride derived from the constituent of a marine fish for activating the PI3K/ERK/CREB signalling pathways in PC12 cells. Int J Mol Sci.

[68] El Omri A, Han J, Kawada K, Ben Abdrabbah M, Isoda H (2012). Luteolin enhances cholinergic activities in PC12 cells through ERK1/2 and PI3K/Akt pathways. Brain Res.

